# Pellagra as It Occurs in Buffalo and Vicinity

**Published:** 1918-07

**Authors:** Grover W. Wende

**Affiliations:** Buffalo, N. Y.


### BUFFALO MEDICAL JOURNAL

Yearly Volume 73 JULY, 1918 Number 12

### ORIGINAL ARTICLES

**The right is reserved to decline papers not dealing with practical medical and surgical subjects, and such as might offend or fail to interest readers. Contributors are solely responsible for opinions, methods of expression and revision of proof.**

### Pellagra as it Occurs in Buffalo and Vicinity

By GROVER W. WENDE, M. D., Buffalo, N. Y.

(Continued from June issue.)

Case No. 9. B., Scotch Canadian housewife; married 15 years, a multipara, aged 39. Born in Merritton, Ontario, Can., where she had always lived. The family history was negative other than that the mother died at 55 from cancer. The personal history showed the patient to have had whooping cough; she was otherwise well and healthy until 1913. Menses, always irregular, appeared during the fourteenth year. She was operated in 1913 for "tumors" since which time she had lost twenty-three pounds in weight. Her digestion always had been fair and she never had suffered from diarrhea. In general she had always felt stronger in winter than in summer. A skin eruption had appeared on the backs of the hands shortly before her operation in 1913, which eruption lasted about three weeks. The patient's environment always had been excellent; she had travelled but little and then never far. She always had been well-fed; corn had entered into her diet only in very moderate amounts. She never had used alcohol; her habits had been excellent.

On August 26th, 1916, the patient sought relief for a skin eruption that came on about the first of August 1916.

Upon examination the nervous system as well as the mental state presented nothing abnormal. The oral, vaginal and rectal mucous membranes were exceedingly red; she complained of abdominal pain; she also gave a history of periodical attacks of diarrhea which often obliged her to go to bed for a short time.

The backs of her hands presented a brownish-red, rough and desquamating chronic dermatitis. A conspicuous and highly characteristic feature was the termination of the inflammation in a well-defined margin upon the wrists, producing the so-called "pellagrous glove." There was no other skin involvement when the patient came under observation.

Examination of the blood showed the hemoglobin to be 77 per cent; the red cells 4,100,000; the white cells 9,600; the leucocyte differential count to be polynuclears 60%; small lymphocytes 24%, large lymphocytes 8%, mononuclears 6% and eosinophiles 2%.

Her physician two months later, October 1916, reported her death; he stated that upon her return home she was confined to her bed; gradually grew weaker; developed a diarrhea with as many as 10 to 20 stools a day. He specially emphasized the dullness and apathy of her mind, and the peculiar expressionless appearance of her face.

Case No. 10. Reported through the courtesy of Dr. Arthur W. Hurd, Superintendent of the Buffalo State Hospital, to whom I am indebted for this history. M. V. S., single white, female American school teacher, aged 63. Born in the State of New Jersey; she had resided in Buffalo upwards of 40 years, graduated from the Buffalo Normal School and subsequently taught in the public schools; she was only absent from Buffalo upon vacation trips.

Family history was negative. Personal history shows that the patient had had no serious illness. Her environment had always been excellent and her habits had been good; she did not use alcohol in any form. Her diet was varied and plentiful with no mention of corn as a special item. Up to 1906 at age of 50, she was extremely particular about her person, her dress and her food, but subsequent to this date it was noted that a gradual change began until she finally preferred, relished and ate heartily of food that was more or less spoiled and which her friends described as "not fit for a dog to eat;" and, until she became careless as to her person and her dress. About this time, 1906, she began to complain of intractable "stomach, bowel, kidney and liver trouble," for which she took much medicine; she also gradually became dependent, self-centered, suspicious, mistrustful and unbelieving. She was constantly seeking relief from her ill-defined disease by frequent changes of doctors, by short periods in sanitariums where she complained that needles were put in her arms and chloroform sprinkled about; these complaints indicate neuralgias. Friends had noticed that the patient's face and particularly her hands had taken on a grimy appearance as if unwashed. The patient was committed to the hospital as an insane person August 28th, 1916, and died February 28th, 1917.

On admission she complained that chloroform was being put on her at night to make her sick and that something was put in the enemas to make her constipated, and that she could not eat for she felt there was no room in her stomach for food and that she felt tired and overworked and did not sleep well.

Examination of the lungs and the heart was negative; the blood pressure gave a systolic reading of 150 m.m., and a diastolic of 100 m.m.; there was no palpable arterio-sclerosis nor lymph nodes. Teeth were unclean and in poor condition; the tongue was coated; the mucous membranes were of good color. The thyroid was not enlarged; there were no tremors nor twitchings. The reflexes were normal. The eyes were normal. The complexion was dark, the skin and subcutaneous tissue showing senile changes. The urine was negative except for a few hyaline casts. The Wassermann blood serum test was negative. The temperature, respiration and pulse were normal.

Following admission the patient became depressed, anxious, worried, contrary, stubborn, resistive, disagreeable and sloven. She did not want to eat; she had to be spoon-fed; she complained that her mouth was full and her stomach and bowels closed. She constantly worried about her bowels and picked at her anus; in December 1916, a diarrhea developed, the stools being very offensive.

In December 1916 although a grimy condition previously had been noted, the backs of the hands became a dark cherry red; on each wrist was an irregular inflamed patch rough and reddish-brown in color; the patches gradually extended down the hand and fingers and up the forearm; they became considerably pigmented and scaly; desquamation occurred between the fingers with the production of raw areas discharging a serous fluid. An unusual skin manifestation was the involvement of the palms which presented a thickened, indurated, darkened, fissured and scaly appearance; patches appeared on the buttocks, on the inner sides of the thighs near the knees and on the dorsal portions of the feet. Another unusual manifestation was the involvement of the soles of the feet in a manner similar to the hands. Later the dermatitis attacked the whole of the right ear and the neck; the involved areas became pigmented, scaly and violaceous in color. The skin of the chin and lips became coarse, rough and grimy with the pores much dilated. The facial expression was one impossible to describe, unlike any noted in connection with other diseases causing suffering and distress. The skin of the entire face was involved, in a spotted appearance. All the involved areas showed evidences of certain changes which indicated that they were repeatedly subjected to an active inflammation; they had assumed different color schemes in different locations; the manifestations of the dermatitis on the different parts of the body varied from time to time.

About 48 hours preceding death the patient developed sudden weakness, labored breathing with a temperature of 106, respirations 40 and pulse 140. The temperature was 95 some hours before death took place.

Autopsy showed the skin conditions as just described. There was thickening and opacity of the intracranial blood vessel trunks; there was no atheroma of the aorta. In the intestines the mucous membrane of the ileum showed several congested dime to half-dollar sized areas, and some patches of atrophy, while that of the colon showed a rather diffuse dark red coloration. The liver showed marked passive congestion. The retro-peritoneal lymph glands were somewhat enlarged. Otherwise the gross appearance was negative.

The microscopical examination of the tissues of the heart showed moderate pigmentation of muscle fibres and slight coronary sclerosis. There was very slight fat infiltration of the liver with some necrosis, and a deposit of pigment around the central vein. The kidneys showed marked chronic parenchymatous nephritis with moderately severe arteriosclerosis. The adrenals had patches of intense congestion. The intestines showed superficial ulceration and patches of hypertrophy of lymphoid and endothelial elements resembling the lesions of typhoid.

In the medulla there were a few blackened fibres (by Marchi) in the pyramids. The medulla and cord R. P. C. L. (by Alzheimer-Mann method) showed a few fibres having early degenerative changes. There was no evidence of tract degeneration in the medulla or cord. There were no senile plaques but much disintegration of intra-cellular neurofibres, especially of the Betz cells.

Left frontal 1. (haematoxylin and eosin). The pia showed only slight if any thickening but the vessels were sclerosed with hypertrophied media; the smaller vessels showed hyalin degeneration. The pia contained dark brown striates and masses of pigment free in its meshes and some in the cytoplasm of the Gitter cells; in some places there were large branching fibroblasts, completely filled with pigment and staining a very dark green with toluidin blue.

The cortex showed a few corpora amylacea in the superficial layer and there was capillary fibrosis.

There was no infiltration of the pia or cortex. There was a moderate satillitosis of the nerve cells especially of the deeper layer; the pyramids were dark and somewhat cloudy.

Left paracentral lobe—Pin-like left frontal 1, perhaps less pigmented. In the cortex was found an enormous number of corpora amylacea in the upper layer. There was very marked satellitosis. There was axonal alteration on left paracentral lobe and right paracentral lobe. For the most part the Betz cells did not contain a great amount of pigment.

Right paracentral lobe (Marchi). A few blackened fibres in the marrow. Lipoid substance in nerve cells and in perivascular spaces and in vessel walls less intensely black. Some similar granules in superficial cortical layers in connection with glia cells and in pia.

Case No. 11. W. V. A., male, half-breed Indian; aged 59, born in New York State; lived in Salamanca, N. Y. He came under observation February 1st, 1917. He had had skin manifestations since December 1916. He had had no mental or nervous symptoms. On examination the mouth and pharynx showed a typical erythema. There was no complaint of the abdominal organs although there had been marked distention for a year. The Wassermann blood test registered four plus. A persistent subacute dermatitis typically pellagrous occupied areas on the face, the neck, the arms and the hands while lesions were present upon other parts of the body. When last heard from the patient had steadily lost weight and still had the skin manifestations.

Case No. 12. R. L., white American housewife; aged 40; born in Liberty, Pa., and lived in Buffalo except for occasional short excursions.

The family history contained the fact that the husband had spinal syphilis otherwise it was negative. The personal history showed patient had had typhoid at 12 years; also pneumonia and the usual children's diseases; she aborted her first pregnancy. During 1916 she had a poor appetite; also loose frequent bowel movements following previous chronic constipation; her sleep was much disturbed; her menstruation quite irregular. Her habits were good; she took no tea, coffee or alcohol. Her diet during the summer season contained much green corn. As she lived most of her life on a farm her environment was good.

The patient entered the Buffalo General Hospital March 28th, 1917, complaining of diarrhea, vomiting, loss of weight and of strength, epigastric pain, burning in the mouth and soreness of the gums and the tongue. She dated the beginning of her disturbances from September 1916 when she was suddenly prostrated which attack was accompanied by nausea, vomiting and diarrhea. The diarrhea had persisted to the extent of from five to fifteen fluid, mucous-like stools daily; she had vomited at least once a day always preceded by gastric pain and nausea. These symptoms were accompanied by a persistent burning pain in tongue, mouth and stomach. At about the same time (September 1916) patches of pigmentation of the skin appeared on the wrists, elbows and face; at first of a bright-red hue these areas became darker from the deposit of pigment and at times were covered with scales.

Examination on admission demonstrated general emaciation and weakness; the lungs, heart, abdomen and pelvis, the nose, eyes, ears and teeth, presented nothing abnormal that would explain the condition of the patient.

In the mouth and pharynx the entire mucosa was found to be of a bright-red color, the gums showed slight ulceration and the tongue was also rough and sore; there was slight palpatory tenderness over the epigastrium. The rectal and colonic mucosa was markedly red and congested; the blood vessels of which were injected while areas of erosion were found above the rectal valves, and the colon contained considerable fluid. The mental state was one of sluggish activity and fear of imposing catastrophe. The expression was dull and mask-like as though the patient had forgotten how to smile. The skin manifestations from which a positive diagnosis was made, were a sharply defined dollar-size patch of desquamating violaceous red hued dermatitis in each precubital space. Upon subsequently exposing the patient to the direct rays of the sun for a few days, there developed a chronic red colored dermatitis covering the face and the backs of the forearms and hands that varied in intensity and extent from time to time.

The temperature range on the day of admission was 100 to 102; it kept at about these degrees during the three months that the patient lived in the hospital being as high as 104 in the mouth and 106 in the rectum two weeks before death, but it was normal (98 to 98.6) on the day preceding her death. The pulse was 130 on entrance and continued frequent the highest record being 160 three weeks before death. Urinalysis, otherwise normal constantly showed the presence of considerable albumen, a few red blood cells and an occasional finely granular cast; the phenolphthalin functional renal test was 45/80 of the normal permeability. the blood examination made on admission showed hemoglobin 85%, red cells 4,310,000, white cells 6,700; differential count,—polynuclears 64%, large lymphocytes 16%, small lymphocytes 18%, and large mononuclear lymphocytes 2%. Blood Wassermann test was negative. Roentgenologic examination showed marked splanchnoptosis with gastric retention of bismuth for six hours; and probably pyloric ulcer. Analysis of the expressed gastric contents removed on passing the tube 23 inches, fifty minutes after an Ewald meal showed the quantity to be 100 c.c.; free acid to be increased from the organic acids as free hydrochloric acid was absent; a large amount of mucous present and there was evidence of very little digestion of the test meal; the presence of macroscopic blood was confirmed by the guaiac test. The expressed contents of the fasting stomach showed a quantity of 70 c.c. of fluid with an acid reaction; a total acidity of 105, with the absence of free hydrochloric acid and the presence of lactic acid; and a large amount of mucous. The presence of blood was demonstrated by both the benzidine and the guaiac tests.

For from seven to ten days preceding death the patient was semiconscious emitting loud screams at frequent intervals during which attacks she became cyanotic; occasionally severe convulsions took place; there was involuntary passing of both urine and feces. Death occurred June 21st, 1917.

No autopsy.

Case No. 13. G. B., white American female; aged 27; born and lived in Austin, Pa. First seen June 5th, 1917. In March 1915, the patient began to have abdominal distress about one-half hour after meals frequently accompanied by vomiting. She lost weight and became weak. Gastric ulcer was diagnosed for which a gastro-enterostomy was done at Austin, Pa., on February 7th, 1916. Ten days later on account of the severity of the symptoms, her appendix and an ovarian cyst were removed. Up to May 15th, 1916, the patient was in a fair physical condition, able to eat and retain solid food although she was having seven to eight stools a day. After May 15th, 1916, she again began to vomit after meals. On May 16th, 1917, the gastro-enterostomy was freed and a pyloroplasty was done. At this time a scaly pigmented skin eruption was present.

Upon physical examination the patient showed some mental weakness. The tongue was slightly inflamed and swollen while the oral mucous membrane showed marked redness. There was general tenderness over the abdomen. The skin of the face, the backs of the hands and the forearms presented the characteristic dermatitis of Pellagra in its chronic phase.

Case No. 14. H. J., American born, colored male, age 43.

Residing in Buffalo when seen June 1st, 1917. He had been ill for seven weeks with vomiting and diarrhea; there had been progressive loss of weight. Examination showed an unusually severe oral inflammation with marked salivation and with great difficulty in both talking and swallowing. The mucous membrane showed marked redness with denuded patches covered with exudate. There was present an acute dermatitis symmetrically distributed upon the dorsa of both hands and both elbows, occupying the sites common to this phase of Pellagra. Although the patient's Pellagra subsided he was quite weak for three months. A recent inquiry shows that he is now living in Trenton, New Jersey.

Case No. 15. W. O., white American female, age 43, resides in Buffalo. She came under observation June 7th, 1917. The patient described an attack similar to that for which she asked consultation as having occurred some two years earlier. The present attack began in January 1917, with an indigestion accompanied by vomiting and with intense paroxysmal abdominal pain which usually came on at night; she was constipated. She presented no other nervous symptoms than those due to habitual loss of sleep. Upon examination there was found a dermatitis typical of Pellagra, limited to the backs of both hands, dark and unlike any other skin eruption, deeply pigmented in typical areas. The affected areas were thickened and fissured, and accompanied by burning sensations. Under the use of the Roentgen rays the thickening of the skin disappeared while the redness and pigmentation continued unabated during the period that the patient was under observation. Today the patient shows slight improvement.

Case No. 16. H. J., male, white American farmer, aged 46; married and the father of six children. Born in the neighborhood of Brocton, New York, and always lived in Western New York. Family history was negative. Personal history was negative. The patient's surroundings had always been good; his diet had always been generous and varied. He had never used alcohol nor had he been exposed to other poisons such as lead and phosphorous.

The patient complained of skin and mouth trouble and of extreme weakness. The patient stated that two or three weeks prior to April 15th, 1917, he had had considerable trouble with his hands which he attributed to their being "chapped" and for which he applied a salve obtained from the druggist; a breaking out of "blisters" followed; later the skin was shed leaving the desquamating surface seen on coming under observation. Four days after first noticing the trouble with the hands swallowing produced considerable pain in the tongue as well as of the entire lining of the mouth. The patient was emaciated. He showed an unhappy, anxious expression. The mucous membrane of the lips, gums, soft palate and cheeks and in fact everywhere else as far as examination was possible was a fiery red. The lower incisor and bicuspid teeth showed the presence of pyorrhea alveolaris. The skin of the dorsum of both hands from the tips of the fingers to above the wrist joints was symmetrically thickened, roughened, wrinkled and covered with fine scales while the natural furrows were exaggerated; the line of demarcation from the healthy skin was very sharp. An affected area extended entirely around the wrists. The only other part of the surface of the body involved was the face in the region of the nose where the patches formed wreath-like figures in a symmetrical manner.

His temperature was 100; pulse, 120; respiration, 24. His blood pressure was 100 m.m. systolic and 50 m.m. diastolic. His blood examination presented nothing remarkable;—the count showed the red corpuscles to be 4,200,000; the leucocytes, 7,000; the hemaglobin, 70%. The gastric contents gave free HCL, 37; a total acidity of 60; there was no occult blood. The urine examination was negative. The Wassermann blood test was negative. The ophthalmoscopic examination was negative. A Roentgenographic examination of the intestinal tract gave nothing definite. The patient died in October 1917, at which time he had developed a condition corresponding to an acute uremia accompanied by a terminal diarrhea. No autopsy.

Case No. 17. X., male, married, white American, aged 43. Born in Addison, New York, where he has always lived except during his attendance at school and short vacations. Family and personal history are negative. His environment was always good. His diet generous and varied except when "drinking." His habits were fair except for the abuse of alcohol. He was considered a heavy drinker for years; he made many efforts to stop trying the different alcoholic cures but soon returning to the habit; his last attempt to quit was made in the early spring of 1917, when he abstained until the first of June; an accident happened at this time to a member of the family and he again started to drink heavily, often taking as much as a quart or more a day during the four months following the accident.

In October 1917, he noticed a peculiar odor to his feet which bathing did not correct; subsequently they became inflamed and swollen; simultaneously, ulcers appeared in his mouth. A persistent nausea came on; a diarrhea appeared with from three to fifteen movements during the night and morning; the stools were black and foul smelling. He continued to drink heavily. He was obliged to give up his work on account of weakness; he went to a sanitarium for three weeks.

Examination on December. 30, 1917, showed the patient to be nervous and apprehensive about his condition. His hair to be dry and lustreless. The skin about his mouth presented a peculiar erythematous condition with slight scaling. The lips were swollen, cracked and bleeding. The mucous membrane of the mouth was fiery red; the tongue was swollen and deeply furrowed; the gums were spongy, presenting an appearance resembling "lead line." The dorsal surface of both hands was covered with a desquamating dermatitis of dark color and many bullae; the invaded areas ended abruptly at the wrists. The toes, the dorsum, and, part of the plantar surfaces of both feet presented an acute inflammation with considerable desquamation. The patient did not complain of itching but of a hypersensitive feeling. The rest of the skin appeared normal.

From a communication dated January 18, 1918, 20 days after the ingestion of alcohol had been entirely stopped, his condition improved in a very remarkable manner. His nervousness and his extreme apprehension entirely disappeared; his mental state readjusted itself so that he was nearly normal. His bowels became normal. The skin manifestations disappeared leaving but a slight disfiguration.

Case No. 18. H. A., white American female, age 27. Residing in Bradford, Pa. The patient came under observation February 20th, 1918. She suffered from pains in the lower extremities. Upon physical examination the characteristic pellagrous stomatitis, vaginitis and proctitis were found. A symmetrical erythematous eruption was present upon the dorsa of both hands, upon both elbows, upon both knees and upon the face. As the disease was subsiding, it presented the characteristic inflammatory dermatitis of this period. This patient seen but once and at so recent a date, seemed to have that type of Pellagra in which a prognosis was very unpromising.

Case No. 19. M. E. P., white female child, aged two years and eight months. Patient's home is in Montgomery, Alabama. The patient was first seen in August 1917. Her mother stated that her year old child had died in 1916 from the same disease and that she had brought the little patient North with the idea that she would receive benefit from the change. The patient had been seen at her home by Dr. Goldberger who made a diagnosis of Pellagra. The child appeared to be in good health; there were present no intestinal symptoms of the disease. She had a dermatitis characteristic of Pellagra on both hands, on both arms from the shoulders to the elbows, upon the dorsa of both feet and upon the neck. The lesions of the hands were darkly pigmented. The collarette was typical. The progress of the disease was favorably influenced by the trip North for it had commenced to disappear. This case although not indigenous to Western New York is included because of its general medical interest.

### CONCLUSIONS.

Of these nineteen cases of Pellagra that have come under my observation since 1910, eighteen were indigenous to Western New York, or the immediately adjacent territory, and one was from the Southern States. In the past eight years, ten came under observation for the first time during the seven years 1910-1917 and nine or about one-half during the year 1917-1918. The apparent increase in the occurrence of Pellagra as shown by these figures is supported by the mortality statistics for the State which records twenty-seven deaths from Pellagra during 1917 as against one in 1912. All of which goes to show that Pellagra is on the increase in New York State. Of the nineteen cases here reported, nine have died, eight are alive and the whereabouts of one is unknown. Of those yet alive two have shown no evidence of the disease during the past two years and two are now inmates of insane asylums.

From my statistics it is evident that Pellagra is no longer a rare disease in New York State but is rather one of comparative frequency. Notwithstanding the large literature upon the subject, medical men are not as yet readily or promptly recognizing typical cases of Pellagra. When it is appreciated that those cases falling under my observation have done so because of the skin manifestations present and that the skin manifestations were so typical as to make the diagnosis unquestioned, then it also must be more than suspected that many cases of Pellagra offering no typical skin manifestations are occurring and the diagnosis is being missed. I would suggest that the incidence of Pellagra be seriously considered when a patient gives a history of periods of otherwise unaccountable ill health, with complaint of vague stomach troubles, with vague nervous symptoms and with greater or less mental depression and especially so if these symptoms are accompanied by uncontrollable diarrhea. It must be remembered that it is possible to have Pellagra without the typical skin manifestations although there is usually present at some time early in the course of the disease a dermatitis of varying duration. The history of the occurrence of a slight skin manifestation coupled with the above symptoms makes the diagnosis almost certain in the event of the nonoccurrence of those typical lesions upon which today a positive diagnosis solely depends.

While the etiology of Pellagra is not yet positively determined the facts bearing upon the solution of this problem that from time to time appear make it probable that a definite cause will be determined in the near future. At present the parasitic and the dietetic theories hold the stage with evidence accumulating favorable now to one and then to the other.

In the series of cases here reported the following facts stand out clearly: The geographical occurrence is very similar to that of hookworm disease and of infantile paralysis, both of which are considered parasitic, in that these cases have cropped out in various localities without apparent rhyme or reason; hence it would seem that Pellagra must have a like causation. The rapid increase in the incidence of the cases since 1907 favors the parasitic and opposes the dietetic theory; the diets of the patients were practically the same from childhood, and were never restricted in either quantity or quality; while with some the diet was especially varied and generous. In none has there been even a suggestion of the poverty upon which other observers seem to dwell as at least an activating cause. The ingestion of very great quantities of alcohol by five of the patients would seem to favor the dietetic theory of the influence of an unbalanced ration. The peculiar dietetic idiosyncrasy of some of the patients also would seem to support the dietetic theory. The weight of the evidence gleaned from these cases would seem to favor the position that a parasite is the causative factor.

### BIBLIOGRAPHY.

John Gray, Amer. Journ. Insan., 1864-1865, Vol. XXI, pp. 223-227.

Samuel Sherwell, Journ. of Cut. and Ven. Dis., 1882-1883, I, 142-145.

Samuel Sherwell, Trans. Amer. Derm. Assn., 1902, p. 76.

Geo. H. Searcy, Journ. Amer. Med. Assn., 1907, Vol. XLIX, p. 37.

Edward J. Wood, Journ. Amer. Med. Assn., 1909, Vol. LIII, p. 274.

J. W. Babcock, Jour. South Carolina Med. Assn., Nov. 1908.

A. Caccini, Med. Record, March 11, 1911, p. 428.

Grover W. Wende, Buffalo Med. Journal, May 1911, p. 193.

F. C. Robbins, Med. Record, Jan. 11, 1913.

Joseph Spangenthal, Buffalo Medical Journal, April 1915.

W. W. Wright, State Hospital Bulletin, Vol. VII, Feb. 1915.

George M. MacKee, Journ. Cut. and Ven. Dis., May 1915.

Wise & Lautman, Journ. Cut. and Ven. Dis., Nov. 1915.

J. M. Winfield, New York Med. Journal, Vol. CII, 1916, p. 1078.

Alfred Potter, Hospital Bulletin, Dept. of Public Charities of the City of New York, April 1917, p. 130.

C. Lombroso, Gaz. Med. Lomb., 1875, No. 38.

Louis W. Sambon, Brit. Med. Journ., 1905, Vol. II, p. 1272.

Alessandrini and Scala, Amer. Med. Assn., Sept. 5, 1914, p. 868.

Joseph Goldberger, United States Public Health Service, Nov. 17, 1916.

E. W. Perdue, American Medicine, 1917, p. 180.

471 Delaware Avenue.

---

**Medical vs. Surgical Treatment of Cancer.** Dr. Frederick Dugdale, of Boston, Mass., in the Charlotte Medical Journal, deplores the present lack of attention to therapeutics in treatment of cancer. Surgery is often only palliative, and frequently impossible. When metastases occur a treatment is needed that will reach all parts of the body through the circulation. The "Dugdale treatment"—specially prepared creosote combined with hydrocarbon and essential oils—is not offered as a specific nor as a substitute for justifiable surgery, but it does possess the desired bactericidal and germicidal properties and does not disturb the stomach. Since 1833, when Reichenbach first used creosote in treating tuberculosis, it has been employed by many clinicians for this disease and for cancer. Dugdale began using it for cancer in 1903, following the formulas of Lepin, Picot and Leo. His present treatment is dispensed in ampoules and administered by injection, by mouth, or as an external application to ulcerated areas. He advises its use in inoperable cases and as a surgical adjunct before and after operation. Dugdale claims that by its use inoperable cases have become operable and that some cases have resulted in clinical cures, without recurrences for periods varying from one to three years.

---

"**Purgation Means Perforation.**" Thaddeus E. Wilkerson of Raleigh, N. C., Memphis Med. Mo., Nov. 1917, reports 4 cases of ruptured appendix from the injudicious use of cathartics. The warning is timely, though it may be questioned whether the rupture is due to the purgative drug so much as to neglect of necessary operation.

